# BET-directed PROTACs in triple negative breast cancer cell lines MDA-MB-231 and MDA-MB-436

**DOI:** 10.1007/s10549-024-07403-w

**Published:** 2024-06-19

**Authors:** Maryana Teufelsbauer, Sandra Stickler, Marie-Therese Eggerstorfer, Dennis Clyde Hammond, Gerhard Hamilton

**Affiliations:** 1 Clinics of Plastic and Reconstructive Surgery, Medical University of Vienna, Vienna, Austria; 2 Institute of Pharmacology, Medical University of Vienna, Vienna, Austria; 3 Center for Breast and Body Contouring, Grand Rapids, MI 49546 USA

**Keywords:** Triple negative breast cancer, BET, PROTAC, ARV-771, MZ1, HER2

## Abstract

**Purpose:**

This study aims to find whether the proliferation and migration of triple negative breast cancer (TNBC) cell lines can be reduced by treatment with bromodomain and extra-terminal domain (BET) inhibitor JQ1 and BET protein targeting chimeras (PROTACs) ARV-771 and MZ1.

**Methods:**

Cytotoxicity tests, scratch migration assays and western blot proteome profiler arrays for protein expression of cancer-related proteins were used to evaluate the impact of a BET-inhibitor and two BET-directed PROTACs on cell viability, migration and on protein expression.

**Results:**

JQ1 and the PROTACs MZ1 and ARV-771 significantly inhibited the growth and migration of the KRAS G13D-mutated MDA-MB-231 cells. In this cell line, the PROTACs suppressed the residual expression of ERBB2/HER2, 3 and 4 that are essential for the proliferation of breast cancer cells and this cell line proved sensitive to HER2 inhibitors. In contrast, the effects of the PROTACs on the protein expression of MDA-MB-436 cells mostly affected cytokines and their cognate receptors.

**Conclusion:**

The degradation of BET-protein by PROTACs demonstrated significant anti-proliferative effects. The KRAS-mutated MDA-MB-231 cells belong to the low-HER2 expressing tumors that have a poorer prognosis compared to HER2-null patients. Since first oral PROTACs against tumor hormone receptors are in clinical trials, this mode of tumor therapy is expected to become an important therapeutic strategy in the future treatment of TNBC.

## Introduction

Breast cancer is a leading cause of death among women worldwide and is one of the most prevalent tumors [1]. Gene expression analysis allows for a categorization of breast cancer into three subtypes [2]. The triple negative breast cancer (TNBC) is characterized by a lack of estrogen or progesterone receptor expression as well as of human epidermal growth factor receptor 2 (ERBB2/HER2) [3]. Studies have shown that gene expression in TNBC is frequently linked to the presence of altered Kirsten rat sarcoma virus (KRAS), which is the most frequently mutated oncogene in cancer [4]. Most KRAS mutations are present at the G12 and G13 hotspots of the gene [5–7]. TNBC is associated with the worst prognosis when considering breast cancers with KRAS mutations [8, 9]. It causes approximately 10–15% of all breast cancers, with a 5-years survival of merely 12% in its disseminated stage, due to the lack of effective therapies [10].

In addition to KRAS, an amplification of its downstream target MYC Proto-Oncogene, BHLH Transcription Factor (MYC) is also frequently found in TNBC [11]. The tumor promoting MYC, which is besides MYCN Proto-Oncogene, BHLH Transcription Factor (MYCN) and MYCL Proto-Oncogene, BHLH Transcription Factor (MYCL) part of the MYC transcription factor (TF) family, also impacts proliferation and malignant transformation [12, 13]. The expression of this oncogene is regulated among others by the bromodomain-containing protein 4 (BRD4). BRD4 is an epigenetic reader protein of the bromodomain and extra-terminal domain (BET) family, consisting of bromodomain testis associated (BRDT), bromodomain containing 2 (BRD2), bromodomain containing 3 (BRD3) and BRD4, that is involved in DNA replication, cell cycle, transcription and signaling during stress responses [14, 15]. BET proteins play a crucial role in carcinogenesis by upregulating gene transcription through the interaction with acetylated-lysine portions of histones [16]. BRD4 recruits the positive elongation factor complex (P-TEFb) and initiates the RNA polymerase activity for a host of genes [17–19]. The C-terminal of BRD4 contains a histone acetyltransferase and can bind to MYC resulting in inhibition of BRD4 histone acetyltransferase [20, 21]. BET-inhibitors can be used to manipulate the MYC expression by targeting BRD4, since these inhibitors mimic the acetyl moiety thereby inhibiting the proliferation of cancer cells [22–24]. Besides in hormone-sensitive and in HER2 positive breast cancer, BET-inhibitors have also shown activity in triple negative breast cancer [25]. The BET inhibitors, like JQ1 have shown only little efficacy as single agents [26]. Therefore, BET-directed proteolysis targeting chimeras (PROTACs) have been developed, which exhibit superior activity in comparison to inhibitors [27]. The degradation of MYC partners by PROTACs has proven successful in clinical models for ARV-771, which depletes the expression of MYC by degrading of BET proteins even in castration-resistant prostate cancer and MZ1, which exhibited anticancer effects in acute myeloid leukemia by targeting MYC [28, 29].

Of the two TNBC cell lines used in this study, MDA-MB-231 carries a KRAS G13D mutation, while MDA-MB-436 is a KRAS wild-type cell line [30]. This study aims to investigate the effects of BET-inhibitor JQ1, and PROTACs MZ1 and ARV-771 on TNBC cell lines MDA-MB-231 and MDA-MB-436 in proliferation assays and migration tests. Western blot proteome profiler arrays are used to evaluate protein expression of 84 cancer-related proteins in these cell lines and the impact of BET-inhibitor, PROTACs and KRAS-inhibitor on their expression. Through these arrays and proliferation and migration assays, this study aims to find how the local migration of breast cancer cells can be reduced or inhibited in patients with TNBC.

## Materials and methods

### Cell lines

Breast cancer cell lines MDA-MB-231 (ATCC HTB-26) and MDA-MB-436 (ATCC HTB-130) were obtained from the American Type Culture Collection (ATCC, Manassas, VA, USA). The cells were validated using short tandem repeat analysis and confirmed to be negative for mycoplasma using MycoStrip® tests (InvivoGen, Tolouse, France).

### Cell culture

The cells used in these experiments were cultivated in RPMI-1640 medium (Sigma-Aldrich, St. Louis, MO, USA) supplemented with 10% fetal bovine serum (Eximus, Catus Biotech, Tutzing, Germany) and antibiotics (Sigma-Adrich). Cells were kept in 75 cm^2^ tissue culture flasks (Greiner Bio-One GmbH, Kremsmuenster, Austria) under cell culture conditions (37°C, 5% CO_2_), regularly split by trypsinization and cell numbers counted with a LUNA cell counter (Biozym, Vienna, Austria).

### Compounds

Compounds ARV-771 and JQ1 were purchased from Selleckchem (Houston TX, USA) and MZ1 has been obtained from Boehringer-Ingelheim (openME, Ingelheim, Germany). All compounds were used as 10 mM stock solutions in dimethyl sulfoxide (DMSO).

### MTT cytotoxicity assay

For this assay, 1 × 10^4^ cells in 100 μL medium were distributed into wells of 96-well flat-bottomed microtiter plates (Techno Plastic Products AG, TPP, Trasadingen, Switzerland) and two-fold dilutions of the test compounds were added in triplicate. Assays were performed at least in triplicate. The plates were incubated for 4 days under tissue culture conditions and viable cells were detected using a modified 3-(4,5-dimethylthiazolyl-2)-2,5-diphenyltetrazolium bromide (MTT) assay (EZ4U, Biomedica, Vienna, Austria). Viable cells were detected at 450 nm and values obtained from control wells containing cells and media alone were set to 100% proliferation. Test results were calculated from dose-response curves using the Origin 9.1 software (OriginLab, Northampton, MA, USA).

### Scratch assay

To perform a scratch assay, the breast cancer cell lines were kept in six-well plates (TPP) in 3 mL complete RPMI-1640 medium until confluency was reached. Subsequently, two perpendicular scratches were set to remove the cells using a plastic tip and the cultures were supplemented with DMSO or the respective inhibitor and further incubated under tissue culture conditions. Light micrographs were acquired (magnification 40×) for 3 successive days and scratch areas not covered by the cells were calculated using the ImageJ software (imagej.net, public domain software) for several positions.

### Proteome profiler arrays

A panel of oncology-related proteins was analyzed using the Proteome Profiler Array (ARY026, R&D Systems, Minneapolis, MN, USA) according to manufacturer’s instructions. Untreated controls were compared to a treatment with MZ1 and ARV-771, respectively. Experiments were performed in duplicate. The arrays were evaluated using Quickspot (Ideal Eyes System, Bountiful, UT, USA) and Origin 9.1 software.

### Reactome pathway database

The reactome pathway database (www.reactome.org) was used to analyze proteins with significantly altered expression after treatment with BET-inhibitors MZ1 and ARV-771, respectively. This pathway database allowed for discovery of functional relationships between these proteins and offered insight into pathways effected by treatments [31].

### Statistical analysis

Analysis was conducted with Origin 9.1 software (OriginLab, Northampton, MA, USA). Statistical significance of values of dose-response curves of different cell lines was tested using students *t*-tests and *p* < 0.05 was regarded as a significant difference. Proteome profiler assays were performed in duplicate and the provided reference spots were used to calibrate the individual chemiluminescence intensities. Pixel values were normalized to ensure comparability and statistical significances *p* < 0.05 were calculated by Students t-tests for two independent assays.

## Results

### Cytotoxicity of BET-inhibitors

**Fig. 1:**
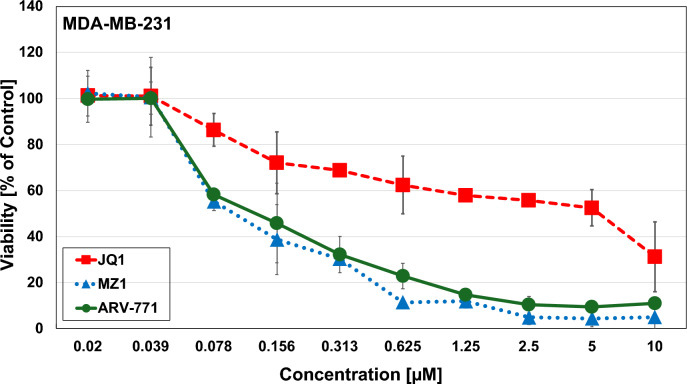
MTT cytotoxicity assays using JQ1, MZ1 and ARV-771 against MDA-MB-231. Data shown are mean values ± SD for 10 2-fold dilutions of the compounds

**Fig. 2:**
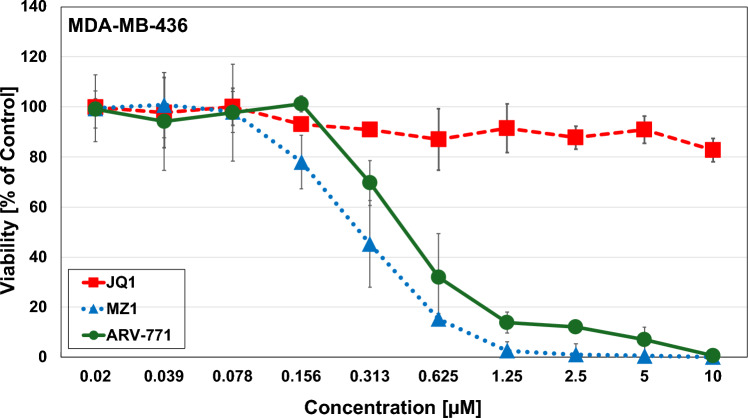
MTT cytotoxicity assays using JQ1, MZ1 and ARV-771 against MDA-MB-436. Data shown are mean values ± SD for 10 two-fold dilutions of the compounds. Effects of BET-inhibitors on cell line migration

The cytotoxic activity of JQ1, MZ1 and ARV-771 was determined using MTT tests for MDA-MB-231 and MDA-MB-436, respectively. The KRAS G13D cell line MDA-MB-231 exhibited chemosensitivity with a half maximal inhibitory concentration (IC_50_ value ± SD) of 5.56 µM ± 0.3 for JQ1, 0.11 µM ± 0.05 for MZ1 and 0.12 µM ± 0.04 for ARV-771 (Fig. 1). JQ1 did not reach IC_50_ concentrations for MDA-MB-436; however low IC_50_-values of 0.24 ± 0.05 for MZ1 and 0.45 ± 0.02 for ARV-771 were detectable (Fig. 2). The BET-directed PROTACs showed higher activity against the KRAS G13D-mutated MDA-MB-231 cells compared to the wildtype MDA-MB436 line.

### Migration assay

**Fig. 3:**
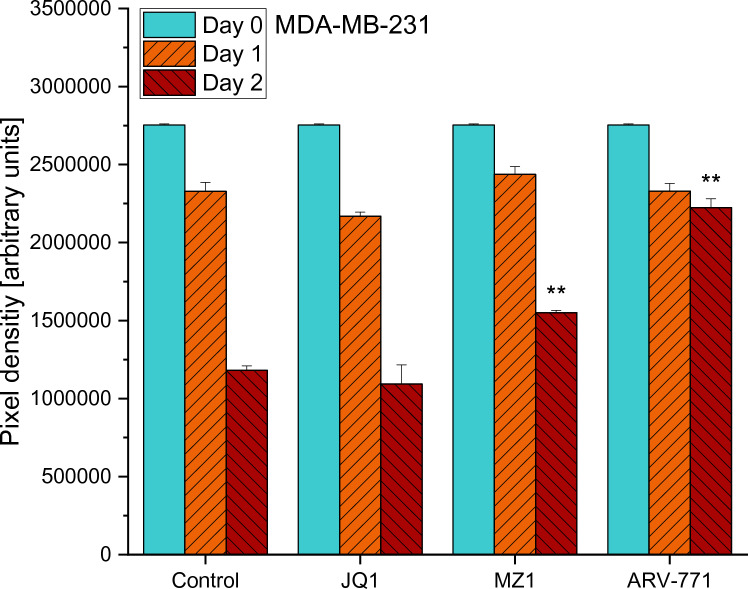
Results of the cell migration assay of MDA-MB-231 cells in a regular medium (Control) and a medium supplemented with either 0.2 µM of JQ1, MZ1 or ARV-771, respectively. Values represent mean values ± SD of the area (arbitrary pixel values) free from migrating tumor cells. Significant *p*-values are indicated by *(*p* < 0.05), **(*p* < 0.01) and ***(*p* < 0.001) and shown for a comparison between treatment and control on the same day

Both MDA-MB cell lines were cultivated in 6-well plates and the migration of the cells into the scratch area was documented using light microscopy for 3 days. The image analysis indicated that 0.2 µM JQ1 did not retard the migration of MDA-MB-231 cells significantly when compared to control values. On day 2, inhibitory effects on cell migration could be observed in treatments with ARV-771 (*p* = 0.0019) and MZ1 (*p* = 0.0039), which both showed a significantly larger clear area than the control did (Fig. 3).

**Fig. 4:**
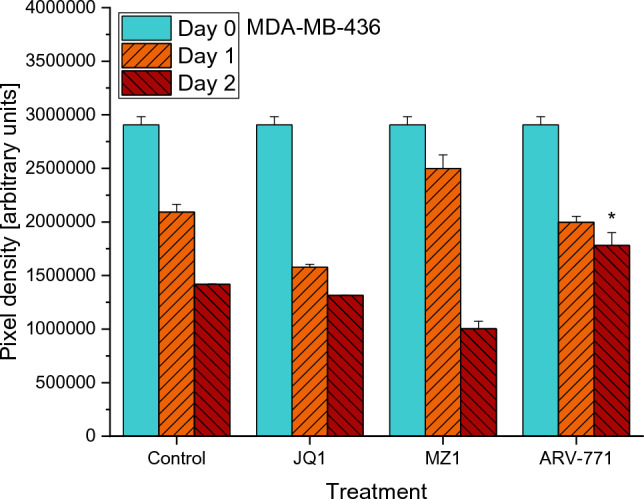
Results of the cell migration assay of MDA-MB-436 cells in a regular medium (Control) and a medium supplemented with either 0.2 µM of JQ1, MZ1 or ARV-771. Values represent mean values ± SD of the area (arbitrary pixel values) free from migrating tumor cells. Significant *p*-values are indicated by *(*p* < 0.05), **(*p* < 0.01) and ***(*p* < 0.001) and shown for a comparison between treatment and control on the same day

Regarding MDA-MB-436, 0.2 µM JQ1 and MZ1 did not exhibit significant effects on cell migration in comparisons between the treatments and the control values of the same days. The only significant effect of a treatment in comparison to the control could be observed in ARV-771 (*p* = 0.0483) for day 2. MZ1 performed significantly better than JQ1 on day 2 (*p* = 0.0097). Moreover, on the second day ARV-771 performed significantly better in inhibiting cell migration than JQ1 (*p* = 0.0299) and MZ1 (*p* = 0.0149) (Fig. 4.).

### Effects of BET-inhibitors on protein expression

**Fig. 5:**
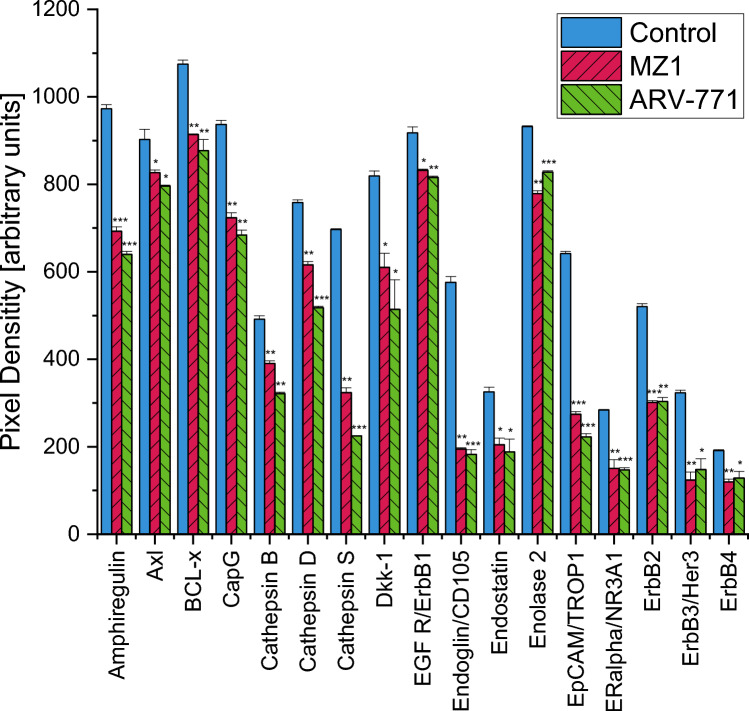
Part 1 of the analysis of cancer-related proteins in MDA-MB-231 following the exposure to 0.2 µM MZ1 and ARV-771. Data represent mean values ± SD. Significant *p*-values are indicated by *(*p* < 0.05), **(*p* < 0.01) and ***(*p* < 0.001). *Axl* AXL Receptor Tyrosine Kinase, *BCL-x* BCL2 Like 1, *CapG* Capping Actin Protein Gelsolin Like, *Dkk-1* Dickkopf WNT Signaling Pathway Inhibitor 1, *EGFR/ErbB1* Epidermal Growth Factor Receptor, *EpCAM/TROP1* Epithelial Cell Adhesion Molecule, *ERalpha/NR3A1* Estrogen Receptor 1, *ErB2* Erb-B2 Receptor Tyrosine Kinase 2, *ErbB3/Her3* Erb-B2 Receptor Tyrosine Kinase 3, *ErbB4* Erb-B2 Receptor Tyrosine Kinase 4

**Fig. 6:**
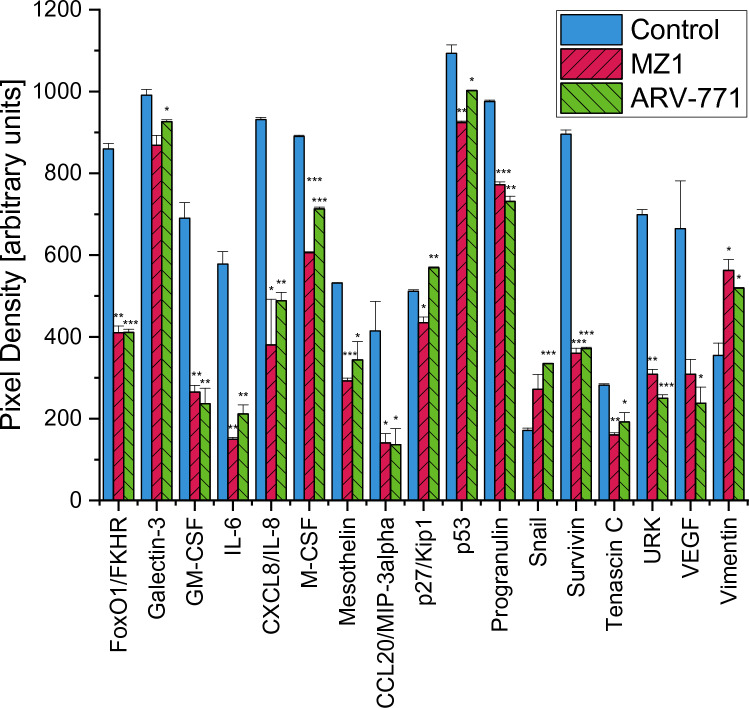
Part 2 of the analysis of cancer-related proteins in MDA-MB-231 following the exposure to 0.2 µM MZ1 and ARV-771. Data represent mean values ± SD. Significant *p*-values are indicated by *(*p* < 0.05), **(*p* < 0.01) and ***(*p* < 0.001). *FoxO1/FKHR* Forkhead Box O1, *GM-CSF* Colony Stimulating Factor 2, *IL-6* Interleukin 6, *CXCL8/IL8* Interleukin 8, *M-CSF* Colony Stimulating Factor 1, *CCL20/MIP-3alpha* C–C Motif Chemokine Ligand 20, *p27/Kip1* Cyclin-Dependent Kinase Inhibitor 1B, Tumor *p53* Protein P53, *Progranulin* Granulin Precursor, *Snail* Snail Family Transcriptional Repressor 1, *Survivin* Baculoviral IAP Repeat Containing 5, *URK* Plasminogen Activator Urokinase, *VEGF* Vascular Endothelial Growth Factor A

The Proteome Profiler Oncology XL detected 32 significantly altered cancer-related proteins after treatment with MZ1, and 34 significantly altered proteins after treatment with ARV-771. Proteins significantly downregulated after both treatments were Amphiregulin, AXL Receptor Tyrosine Kinase (Axl), BCL2 Like 1 (BCL-x), Capping Actin Protein Gelsolin Like (CapG), Cathepsin B, Cathepsin D, Cathepsin S, Dickkopf WNT Signaling Pathway Inhibitor 1 (Dkk-1), Epidermal Growth Factor Receptor (EGFR/ErbB1), Endoglin, Endostatin, Enolase 2, Epithelial Cell Adhesion Molecule (EpCAM/TROP1), Estrogen Receptor 1 (ERalpha/NR3A1), Erb-B2 Receptor Tyrosine Kinase 2 (ErbB2), Erb-B2 Receptor Tyrosine Kinase 3 (ErbB3/Her3), Erb-B2 Receptor Tyrosine Kinase 4 (ErbB4), Forkhead Box O1 (FoxO1/FKHR), Colony Stimulating Factor 2 (GM-CSF), Interleukin 6 (IL-6), Interleukin 8 (CXCL8/IL8), Colony Stimulating Factor 1 (M-CSF), Mesothelin, C–C Motif Chemokine Ligand 20 (CCL20/MIP-3alpha), Tumor Protein P53 (p53), Granulin Precursor (Progranulin), Baculoviral IAP Repeat Containing 5 (Survivin), Tenascin C, Plasminogen Activator Urokinase (URK) and Vascular Endothelial Growth Factor A (VEGF). Additionally, to these proteins, treatment with ARV-771 also significantly decreased Galectin-3. The Cyclin-Dependent Kinase Inhibitor 1B (p27/Kip1) was downregulated after a treatment with MZ1 and upregulated after a treatment with ARV-771. Snail Family Transcriptional Repressor 1 (Snail) and Vimentin were also upregulated with ARV-771, while only Vimentin was upregulated after treatment with MZ1 (Figs. 5, 6).

### Reactome overexpressed pathways analysis

In MDA-MB-231 treatment with BET-inhibitors MZ1 and ARV-771 caused the greatest statistical significance in the same pathways, namely Interleukin-4 and Interleukin-13 signaling, Interleukin-10 signaling, ERBB2 Activates PTK6 Signaling, ERBB2 Regulates Cell Motility, Constitutive Signaling by Aberrant PI3K and PIP3 activates AKT signaling although with differing *p*-values (Tables 1, 2).

**Table 1:** A selection of the significantly altered pathways according to proteins affected by treatment with MZ1 in MDA-MB-231

Pathway name	*p*-value	FDR
Interleukin-4 and interleukin-13 signaling	1.11e−16	2.32e−14
Interleukin-10 signaling	3.20e−14	3.33e−12
ERBB2 Activates PTK6 signaling	1.02e−10	4.65e−09
Constitutive signaling by aberrant PI3K	1.22e−10	4.65e−09
ERBB2 Regulates cell motility	1.41e−10	4.78e−09
PIP3 Activates AKT signaling	1.95e−10	6.24e−09

Results are sorted according to p-value

**Table 2:** A selection of the significantly altered pathways according to proteins affected by treatment with ARV-771 in MDA-MB-231

Pathway name	*p*-value	FDR
Interleukin-4 and interleukin-13 signaling	2.22E−16	9.33E−14
Interleukin-10 signaling	6.91E−14	7.25E−12
ERBB2 Activates PTK6 signaling	1.53E−10	7.05E−9
ERBB2 Regulates cell motility	2.11E−10	8.58E−9
Constitutive signaling by aberrant PI3K	2.26E−10	8.58E−9
PIP3 activates AKT signaling	4.67E−10	1.49E−8

Results are sorted according to p-value

**Fig. 7:**
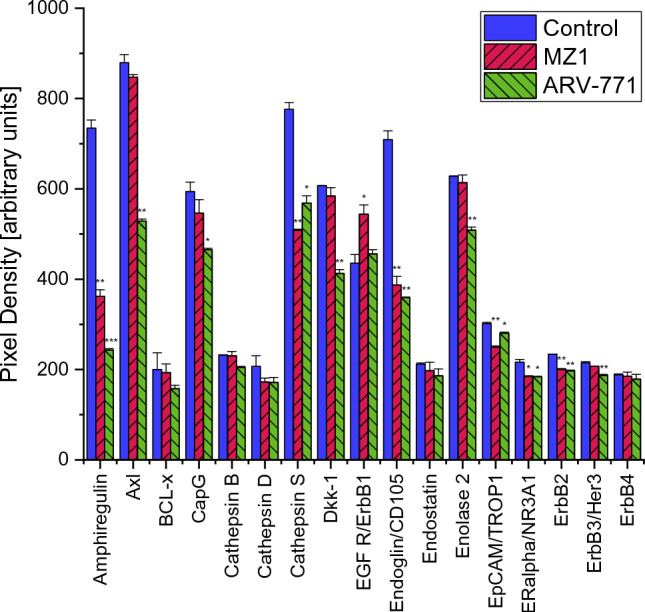
Part 1 of the analysis of cancer-related proteins in MDA-MB-436 following the exposure to 0.2 µM MZ1 and ARV-771. Data represent mean values ± SD. Significant *p*-values are indicated by *(*p* < 0.05), **(*p* < 0.01) and ***(*p* < 0.001). *Axl* AXL Receptor Tyrosine Kinase, *BCL-x* BCL2 Like 1, *CapG* Capping Actin Protein Gelsolin Like, *Dkk-1* Dickkopf WNT Signaling Pathway Inhibitor 1, *EGFR/ErbB1* Epidermal Growth Factor Receptor, *EpCAM/TROP1* Epithelial Cell Adhesion Molecule, *ERalpha/NR3A1* Estrogen Receptor 1, *ErB2* Erb-B2 Receptor Tyrosine Kinase 2, *ErbB3/Her3* Erb-B2 Receptor Tyrosine Kinase 3, *ErbB4* Erb-B2 Receptor Tyrosine Kinase 4

**Fig. 8:**
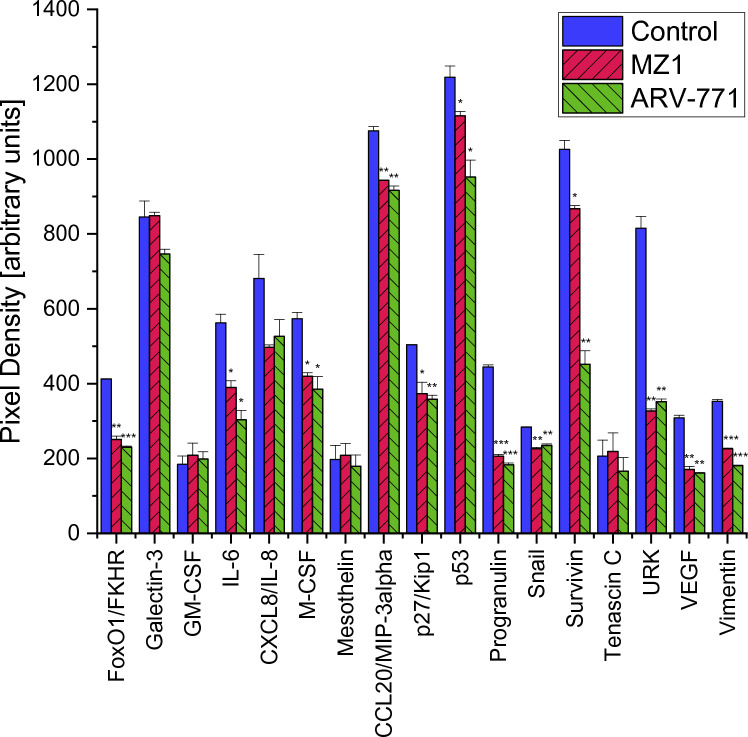
Additionally to the comparison with MDA-MB-231, significant changes in protein expression of MDA-MB-436 are shown in this graph. Part 3 of the analysis of cancer-related proteins in MDA-MB-436 following the exposure to 0.2 µM MZ1 and ARV-771. Data represent mean values ± SD. Significant *p*-values are indicated by *(*p* < 0.05), **(*p* < 0.01) and ***(*p* < 0.001). *HIF-1alpha* Hypoxia Inducible Factor 1 Subunit Alpha, *HNF-3beta* Forkhead Box A2, *MUC-1* Mucin 1 Cell Surface Associated, *Serpin B5/Maspin* Serpin Family B Member 5, *SerpinE1/PAI-1* Serpin Family E Member 1

In MDA-MB-436 a Proteome Profiler Oncology XL detected 25 proteins significantly altered by MZ1 and 29 proteins significantly altered by ARV-771. Proteins significantly downregulated after exposition to 0.2 µM of both PROTACs were Amphiregulin, Cathepsin S, Endoglin, Epithelial Cell Adhesion Molecule (EpCAM/TROP1), Estrogen Receptor 1 (ERalpha/NR3A1), Erb-B2 Receptor Tyrosine Kinase 3 (ErbB3/Her3), Forkhead Box O1 (FoxO1/FKHR), Interleukin 6 (IL-6), Colony Stimulating Factor 1 (M-CSF), C–C Motif Chemokine Ligand 20 (CCL20/MIP-3alpha),Cyclin-Dependent Kinase Inhibitor 1B (p27/Kip1), Tumor Protein P53 (p53), Granulin Precursor (Progranulin), Snail Family Transcriptional Repressor 1 (Snail), Baculoviral IAP Repeat Containing 5 (Survivin), Plasminogen Activator Urokinase (URK), Vascular Endothelial Growth Factor A (VEGF), Vimentin, Hypoxia Inducible Factor 1 Subunit Alpha (HIF-1alpha), Forkhead Box A2 (HNF-3beta), Mucin 1 Cell Surface Associated (MUC-1), Serpin Family B Member 5 (Serpin B5/Maspin) and Serpin Family E Member 1 (SerpinE1/PAI-1). A treatment with MZ1 additionally decreased Epidermal Growth Factor Receptor (EGFR/ErbB1) significantly. While a treatment with ARV-771 also decreased AXL Receptor Tyrosine Kinase (Axl), Capping Actin Protein Gelsolin Like (CapG), Dickkopf WNT Signaling Pathway Inhibitor 1 (Dkk-1), Enolase 2 and B2 Receptor Tyrosine Kinase 3 (ErbB3/Her3) (Figs. 7, 8).

A pathway analysis in MDA-MB-436 after treatment with MZ1 and ARV-771 also featured the same pathways among the lowest *p*-values, namely Interleukin-4 and Interleukin-13 signaling, Constitutive Signaling by Aberrant PI3K, PIP3 activates AKT signaling, PI5P, PP2A and IER3 Regulate PI3K/AKT Signaling and Regulation of gene expression by Hypoxia-inducible Factor at differing *p*-values (Tables 3, 4).

**Table 3:** A selection of the significantly altered pathways according to proteins affected by treatment with MZ1 in MDA-MB-436

Pathway name	*p*-value	FDR
Interleukin-4 and interleukin-13 signaling	1.11e−16	4.31e−14
Constitutive signaling by aberrant PI3K	1.25e−08	6.88e−07
PIP3 activates AKT signaling	2.22e−08	1.07e−06
PI5P, PP2A and IER3 regulate PI3K/AKT signaling	9.23e−08	3.51e−06
Regulation of gene expression by Hypoxia-inducible Factor	1.23e−05	3.57e−04

Results are sorted according to p-value

**Table 4:** A selection of the significantly altered pathways according to proteins affected by treatment with ARV-771 in MDA-MB-436

Pathway name	*p*-value	FDR
Interleukin-4 and interleukin-13 signaling	1.11e−16	4.30e−14
PIP3 activates AKT signaling	2.78e−08	1.53e−06
Constitutive signaling by aberrant PI3K	4.16e−07	1.79e−05
PI5P, PP2A and IER3 regulate PI3K/AKT signaling	2.28e−06	7.98e−05
Regulation of gene expression by hypoxia-inducible factor	1.32e−05	3.82e−04

**Fig. 9:**
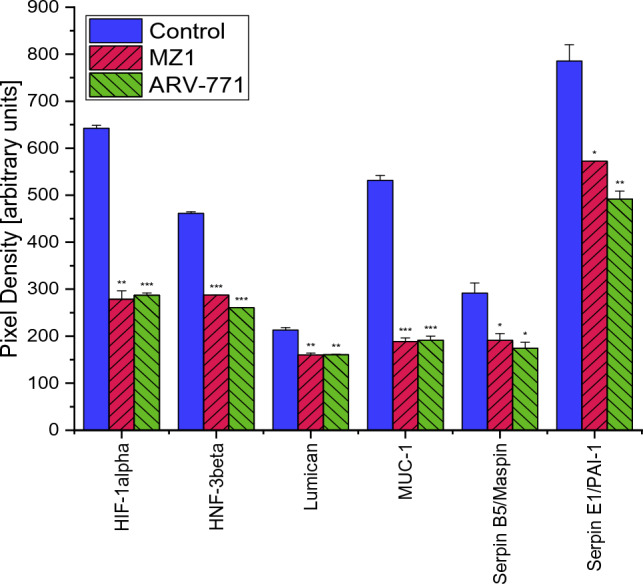
Part 2 of the analysis of cancer-related proteins in MDA-MB-436 following the exposure to 0.2 µM MZ1 and ARV-771. Data represent mean values ± SD. Significant *p*-values are indicated by *(*p* < 0.05), **(*p* < 0.01) and ***(*p* < 0.001). *FoxO1/FKHR* Forkhead Box O1, *GM-CSF* Colony Stimulating Factor 2, *IL-6* Interleukin 6, *CXCL8/IL8* Interleukin 8, *M-CSF* Colony Stimulating Factor 1, *CCL20/MIP-3alpha* C–C Motif Chemokine Ligand 20, *p27/Kip1* Cyclin-Dependent Kinase Inhibitor 1B, *p53* Tumor Protein P53, *Progranulin* Granulin Precursor, *Snail* Snail Family Transcriptional Repressor 1, *Surivivin* Baculoviral IAP Repeat Containing 5, *URK* Plasminogen Activator Urokinase, *VEGF* Vascular Endothelial Growth Factor A

In contrast to MDA-MB-231 the proteins BCL2 Like 1 (BCL-x), Cathepsin B and D, Endostatin, Erb-B2 Receptor Tyrosine Kinase 4 (ErbB4), Galectin-3, Colony Stimulating Factor 2 (GM-CSF), Interleukin 8 (CXCL8/IL8), Mesothelin and Tenascin C were not significantly altered in MDA-MB-436 after an identical treatment with MZ1 and ARV-771. In contrast, the proteins Hypoxia Inducible Factor 1 Subunit Alpha (HIF-1alpha), Forkhead Box A2 (HNF-3beta), Mucin 1 Cell Surface Associated (MUC-1), Serpin Family B Member 5 (Serpin B5/Maspin) and Serpin Family E Member 1 (SerpinE1/PAI-1) were not significantly altered in MDA-MBA-231 but downregulated in MDA-MB-436 (Figs. 4, 5, 6, 7, 8 and 9).

Pathway analysis revealed differences in pathways affected by MZ1 and ARV-771 between MDA-MB-231 and MDA-MB-436. While Interleukin-4 and Interleukin-13 signaling, PIP3 activates AKT signaling and Constitutive Signaling by Aberrant PI3K seemed to be affected in both cell lines, pathways regarding ERBB2, namely ERBB2 Activates PTK6 Signaling and ERBB2 Regulates Cell Motility were only affected in the KRASG13 MDA-MB-231 (Tables 1, 2, 3, 4).

### Effects of HER-2 inhibitors

**Fig. 10:**
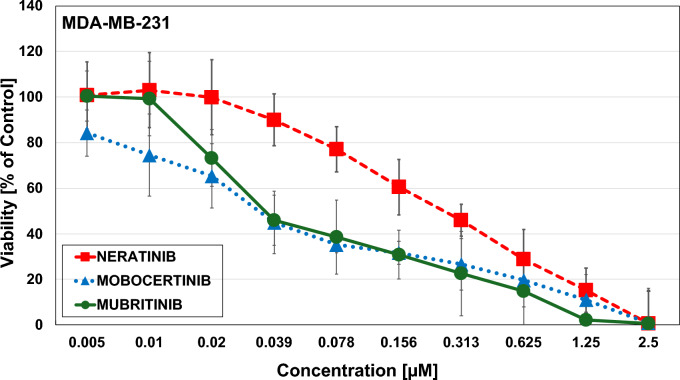
Triple negative breast cancer cell lines MDA-MB-231 after exposure to Neratinib, Mobocertinib and Mubritinib evaluated via MTT cytotoxicity assays. Data shown are mean values ± SD for 10 two-fold dilutions of the compound

**Fig. 11:**
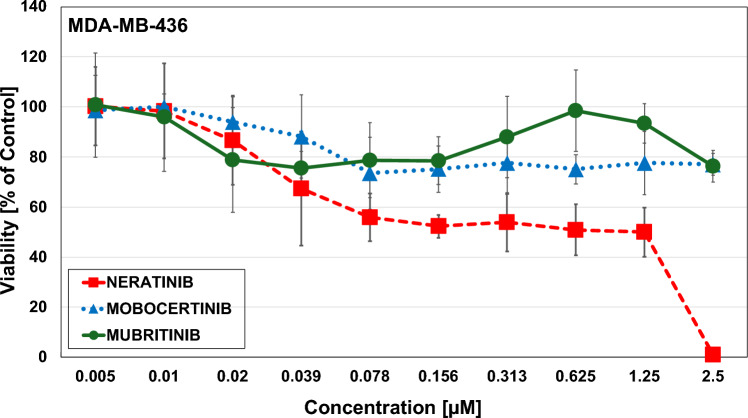
Triple negative breast cancer cell lines MDA-MB-436 after exposure to Neratinib, Mobocertinib and Mubritinib evaluated via MTT cytotoxicity assays. Data shown are mean values ± SD for 10 two-fold dilutions of the compound

The HER-2 inhibitor Neratinib showed an IC_50_-value of 0.2 µM ± 0.08, Mobocertinib 0.013 ± 0,00424 and Mubritinib 0.15 ± 0.007 (Fig. 10) for the MDA-MB-231 cells. For the MDA-MB-436 cell line, no IC_50_-values could be achieved for HER-2 inhibitor Mubritin and Mobocertinib at a starting concentration of 2.5 µM, while Neratinib showed an IC_50_-value of 0.57 ± 0.07 (Fig. 11). Thus, the HER2 inhibitors proved effective in the KRAS G13D line that showed a residual activity of HER2/ERBB2 and ERBB3/4.

### Effects of the EGFR inhibitors Afatinib and Osimertinib

**Fig. 12:**
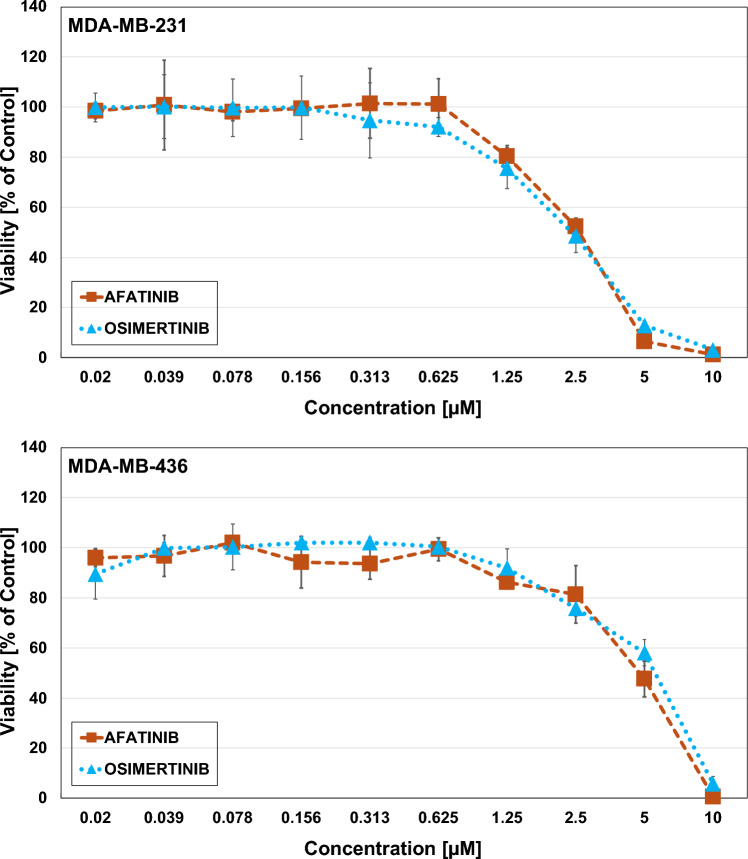
Triple negative breast cancer cell lines MDA-MB-231 (above) and MDA-MB-436 (below) after exposure to Afatinib and Osimertinib evaluated via MTT (3-(4, 5-dimethylthiazolyl-2)-2, 5-diphenyltetrazolium bromide) cytotoxicity assays. Data shown are mean values ± SD for 10 two-fold dilutions of the compound

MDA-MB-231 proofed more sensitive to Afatinib and Osimertinib than MDA-MB-436. The IC_50_-value of MDA-MB-231 with Afatinib and Osimertinib are 2.5 µM and 2.8 µM, respectively, while those of MDA-MB-436 are 4.73 µM for Afatinib and 3.47 µM for Osimertinib (Fig. 12).

## Discussion

The BET-directed agents impaired the proliferation and migration of both TNBC cell lines MDA-MB-231 and MDA-MB-436, with higher effects of the PROTACs compared to the JQ1 inhibitor and higher efficacy against the KRAS G13D-mutated MDA-MB-231 cell line. ARV-771 is a broad-spectrum BET-degrader; whereas MZ1 mainly targets the BRD4 family member. Both degraders ultimately effect the MYC protein that transmits the growth factor/KRAS-triggered proliferative signals in the cell nucleus. The Western Blot Protein Profiler for 84 cancer-related proteins revealed several significant protein alternations in both cell lines. Both BET PROTACs MZ1 and ARV-771 were able to downregulate all ERBB family receptors in MDA-MB-231, which play key roles in activating the Mitogen-Activated Protein Kinase (MAPK) pathway and are connected to the development of many types of cancer [32]. Further, an overexpression of EGFR/ErbB1 and especially ErbB2 is often found in breast cancer [33]. BCL-x is also highly expressed in MDA-MB-231, which could be due to the influence of KRAS G13 on the MAPK/ ERK pathway, therefore leading to an increase in downstream targets, including the anti-apoptotic BCL-x [34]. The MAPK/ERK pathway could further explain the high level of Cathepsin D, since it could be found that an increase of Cathepsin D in human endothelial cells resulted in an activation of ERK 1/2 [35]. BCL-x and Cathepsin D were both significantly decreased by the BET PROTACs used in this study. Due to these significant alternations a decreased function of the MAPK/ERK pathway could lead to a decreased viability and invasion of MDA-MB-231 in viability tests and scratch assays (Figs. 2, 3).

Despite downregulation of proteins associated with the MAPK/ERK pathway, several other significant changes could be discovered. An overexpression of Cathepsin B as observed in MDA-MB-231, is associated with an increased cancer progression, namely growth, tumorigenesis, and invasion, as shown for colorectal cancer [36]. Endoglin was highly expressed in both TNBCs and is in vivo responsible for a cold tumor phenotype by preventing angiogenesis, inflammation and accumulation of cancer-associated fibroblasts [37, 38]. GM-CSF and M-CSF were both highly expressed in MDA-MB-231, while only M-CSF was highly expressed in MDA-MB-436. The expression of these two proteins has been shown to be significantly increased in a study with 54 breast cancer patients compared to control patients [39]. All three overexpressed proteins could be significantly decreased after a treatment with MZ1 and ARV-771. Interleukin-8, which exhibited high expression rates in the control, likely due to a known overexpression in oncogenic KRAS that promotes cell growth and cell migration was successfully downregulated by both BET PROTACs [40]. Further, both treatments resulted in a decreased expression of Mesothelin. A high expression of Mesothelin is associated with KRAS mutations and entails a poor prognosis for treatment [41]. ARV-771 was able to upregulate p27/Kip1 in MDA-MB-231, which is frequently downregulated in human tumors [42]. It can block cell cycle progression and was shown to lead to irreversible growth arrest in several KRAS colorectal cancer cell lines [43].

HER2/ERBB2 overexpression is found in approximately 20% of breast cancers but the standard therapy, the monoclonal HER2 antibody Trastuzumab, fails in one third of the patients [44]. Other monoclonal antibodies such as pertuzumab and margetuximab, as well as trastuzumab-based antibody–drug conjugates can be administered. Trastuzumab constitutes an humanized 4d5 mouse monoclonal antibody that inhibits the ligand-induced HER2 activation [45]. In trastuzumab-deruxtecan (T-DXd), trastuzumab is conjugated to the topoisomerase I inhibitor Exatecan to present a highly specific antibody–drug conjugate, as shown in the DESTINY-Breast04 phase III trial [46]. In contrast to trastuzumab, pertuzumab acts as a dimerization inhibitor of HER2 and margetuximab is a chimeric antibody that binds HER2 and activates immune effector cells via CD16A binding domain [47, 48].

Approximately 50% of BCs, categorized as HER2-negative, have low expression of HER2 with an immunohistochemical (IHC) score of 1–2 and negative results in in situ hybridization [49]. Studies indicate that HER2-low TNBC patients feature more aggressive tumor characteristics than the HER2-null phenotype, including larger tumor size, frequent lymph nodes involvement, higher grade and a worse prognosis (HR [CI 95%] =3.44) with an adverse immune microenvironment [50, 51]. MDA-MB-231 exhibits significantly higher ERBB family member expression compared to KRAS wildtype MDA-MB-436 cells and seems to belong to the HER2/ERBB2 low breast cancer category. The two BET PROTACs suppress the protein expression of the ERBB family members in the MDA-MB-231, that may be responsible for the higher inhibition of the cell proliferation. Functional expression of the ERBB family members in this cell line is demonstrated by the antiproliferative activity of three different HER2/ERBB2 inhibitors that show less activity in the MDA-MB-436 cells. Furthermore, pathway analysis has shown an involvement of PIK3 in good correspondence with the presence of *PIK3CA* mutations in HER2-low tumors [52]. The upstream inhibition of the EGFR receptor using afatinib or Osimertinib failed to exert antiproliferative effects in both MDA-MB cell lines.

## Conclusion

The downstream signal transduction of cell growth factor receptors via KRAS and SOS1 ultimately regulates the phosphorylation and expression of MYC that activates distinct pathways in the cell nucleus leading to malignant proliferation, migration and resistance. Since for the inhibition of MYC no suitable agents are available, MYC can be suppressed by BET inhibitors through small molecular drugs or, more efficiently, through PROTACs. Our results demonstrate that the PROTACs MZ1 and ARV771 significantly inhibit the growth and migration of MDA-MB-231 cells. In good correspondence with the KRAS G13D mutation and the HER2-low status of the MDA-MB-231 cells, the PROTACs suppress the residual expression of ERBB2, 3 and 4 that are essential for the progress of breast cancer cells. In contrast, the effects of the PROTACs on the protein expression of MDA-MB-436 cells yielded a different pattern, mostly affecting cytokines and their cognate receptors. In summary, the degradation of BET-protein by PROTACs demonstrated significant tumor-suppressive effects. Since first oral PROTACs against tumor hormone receptors are in clinical trials, this mode of tumor therapy is expected to become an important in the future treatment of breast cancer.

## Data Availability

Enquiries about data availability should be directed to the authors.
